# *In vitro* characterization of the candidate prebiotic potential of melon and watermelon seeds: strain-specific fermentation and targeted metabolic profiling

**DOI:** 10.1515/biol-2025-1386

**Published:** 2026-10-12

**Authors:** Sengul Teksoy, Lutfiye Yilmaz-Ersan

**Affiliations:** Department of Food Engineering, Faculty of Agriculture, Bursa Uludag University, Bursa, Türkiye

**Keywords:** *Cucumis melo* seed, *Citrullus lanatus* seed, strain-specific fermentability, prebiotic, sugar bioconversion, short-chain fatty acid

## Abstract

This study evaluated water-extractable fractions of melon (*Cucumis melo*) and watermelon (*Citrullus lanatus*) seeds as candidate prebiotic substrates using a controlled *in vitro* monoculture model. Fermentative responses of five probiotic strains were assessed through growth kinetics, pH, prebiotic activity score (PAS), sugar utilization, and lactic acid and short-chain fatty acid (SCFA) profiles. Substrate, strain, and fermentation time, along with their interactions, significantly affected microbial growth and metabolic responses (*p* < 0.01). PAS values demonstrated distinct strain-substrate selectivity: watermelon seed elicited a strong positive response in *B. bifidum* but a negative response in *L. casei*. Despite an overall reduction in sugars at the end of fermentation, utilization patterns differed by strain; *L. acidophilus* was distinguished by fructose consumption, particularly in watermelon seed, and by galactose consumption in melon seed. Metabolite profiles did not directly correspond with growth responses; the watermelon seed-*L. casei* combination was notable for lactic acid production, while the highest total SCFA was generated in glucose medium and among probiotics by *L. acidophilus*. The results indicate that seed fractions do not exhibit a general superiority in fermentation but rather exhibit selective utilization patterns that differ along the axes of strain and metabolic response.

## 1 Introduction

The transformation of by-products generated in the food production chain into nutritional and functional components has become a primary research area at the intersection of sustainable nutrition and the circular bioeconomy. Particularly, peel, seed, and pomace fractions released during fruit and vegetable processing can exhibit a remarkable composition in terms of dietary fiber, carbohydrates, proteins, lipids, minerals, and phenolic compounds, thereby constituting an important resource for the recovery of functional components from low-value waste streams [1], [2], [3], [4], [5]. Within the scope of this approach, the conversion of plant-based by-products into substrates usable by intestinal microorganisms is a particularly noteworthy research axis that combines waste valorization with microbiota-focused nutrition strategies [3], [4], [5]. The International Scientific Association for Probiotics and Prebiotics (ISAPP) defines a prebiotic as “a substrate that is selectively utilized by host microorganisms conferring a health benefit” [6]. This definition requires the evaluation not only of fermentability but also of microbial utilization selectivity, together with demonstrated health benefits for the host. While inulin, fructooligosaccharides, and galactooligosaccharides are among the most comprehensively characterized prebiotics in this field, interest in evaluating complex carbohydrate fractions derived from agro-food by-products as novel prebiotic candidates is increasing [4], [7], [8], [9], [10], [11], [12], [13], [14], [15], [16], [17], [18], [19]. The biological functionality of these substrates depends not only on their capacity to support probiotic growth but also on their selective utilization by different microorganisms, the transformation of carbohydrates during fermentation, and the formation of metabolic products such as lactic acid and short-chain fatty acids (SCFA) [6], [7], [8], [9], [20]. Indeed, ISAPP emphasizes that microbial selectivity is a fundamental element of the prebiotic concept and that metabolites formed by fermentation should be evaluated as functional outputs of the substrate–microbiota interaction.

Melon (*Cucumis melo* L.) and watermelon (*Citrullus lanatus*) are widely consumed members of Cucurbitaceae worldwide, generating significant amounts of peel and seed by-products during processing and consumption [21], [22], [23], [24], [25], [26], [27], [28]. The richness of these fractions in oil, protein, dietary fiber, and various phytochemicals enables their valorization in food and biotechnology applications beyond traditional waste management [2], 21], [23], [24], [25], [26], [27], [28], [29], [30], [31], [32], [33], [34], [35], [36], [37], [38]. Melon seeds have been reported to contain approximately 30–40 % lipid, 15–25 % protein, and considerable amounts of fiber; watermelon seeds constitute a nutrient-dense matrix containing approximately 38–45 % lipid and 25–37 % protein [2], 21], [29], [30], [31], [39], 40]. The formation of a complex plant matrix by the carbohydrate/fiber fractions present in both seeds, together with proteins, lipids, and phenolic compounds, suggests that their fermentative utilization characteristics may differ from pure commercial prebiotics. Therefore, the functional value of these by-products should be evaluated not only through their nutritional composition or antioxidant properties, but also through the substrate–microorganism interactions they form with probiotic microorganisms. Evidence supporting the biological validity of this approach is increasingly strengthening, particularly on other by-products of melon. Gómez-García et al. [41] demonstrated that melon peel flour subjected to *in vitro* gastrointestinal digestion supported the growth of *Lactobacillus/Lacticaseibacillus* and *Bifidobacterium* strains following fermentation with human fecal microbiota, influenced microbiota structure, and was associated with the formation of mainly acetate, propionate, and butyrate. Pérez-Juárez et al. [34] reported that bacterial growth, carbohydrate consumption, and SCFA formation varied according to substrate–microorganism combination during fermentation of melon and watermelon peel flours by *L. brevis*, *L. paracasei*, and *L. rhamnosus*. The fact that previous studies conducted on melon by-products have also evaluated the seed fraction as a fermentative substrate [16] supports the microbial utilization potential of Cucurbitaceae by-products. However, the existing literature has largely focused on peel fractions or total by-products; studies evaluating the selective utilization of melon, particularly watermelon, seeds by different *Lactobacillus/Lacticaseibacillus* and *Bifidobacterium* strains, strain-specific metabolic utilization of carbohydrates, and the concomitant formation of fermentation metabolites through an integrated experimental approach have remained limited. This situation constitutes a significant knowledge gap when considering the distinctive matrix of seeds with high lipid and protein content.

In this regard, the present study aimed to investigate the fermentative properties of water-extractable fractions of melon and watermelon seeds as potential prebiotic substrates, with a focus on strain-substrate specificity. Using *Lactobacillus acidophilus, Lacticaseibacillus casei, Bifidobacterium longum* subsp. *infantis, Bifidobacterium animalis* subsp. *lactis*, and *Bifidobacterium bifidum*, microbial growth was monitored through pH, optical density, and viable cell counts; relative selectivity in substrate utilization was evaluated with the prebiotic activity score. To characterize the metabolic features of the fermentative response, utilization profiles of glucose, fructose, galactose, xylose, and sucrose, as well as the formation of lactic acid and short-chain fatty acids (SCFA; acetate, propionate, and butyrate) were determined. Thus, by comprehensively evaluating microbial proliferation, substrate selectivity, carbohydrate utilization profile, and fermentative metabolite formation, the study aimed to elucidate the strain-substrate specificity in the utilization of melon and watermelon seeds by different probiotic strains and the accompanying fermentative responses.

## 2 Materials and methods

### 2.1 Probiotic bacterial strains

The probiotic strains used in the present study are shown in Table 1. Probiotics were supplied by DSMZ (Deutsche Sammlung von Mikroorganismen und Zellkulturen GmbH, Braunschweig, Germany) and activated at 37 °C under an anaerobic atmosphere generated with an Anaerocult A system (Merck, Darmstadt, Germany). For the second activation, Tryptone Peptone Yeast Extract (TPY) broth for *Bifidobacterium* species, and a De Man Rogosa and Sharpe (MRS) broth for *Lactobacillus/Lacticaseibacillus* species were prepared. TPY broth composed of tryptone (10.00 g L^−1^), glucose (5.00 g L^−1^), peptone (5.00 g L^−1^), yeast extract (2.50 g L^−1^), K_2_HPO_4_·3H_2_O (2.00 g L^−1^), Tween 80 (1.00 g L^−1^), MgCl_2_ (0.50 g L^−1^), ZnSO_4_·7H_2_O (0.20 g L^−1^), CaCl_2_ (0.15 g L^−1^), FeCl_3_·6H_2_O (0.003 g L^−1^), and *L*-cysteine HCl (0.50 g L^−1^). MRS broth included meat extract (10.00 g L^−1^), casein peptone (10.00 g L^−1^), yeast extract (5.00 g L^−1^), glucose (2.00 g L^−1^), Na-acetate (5.00 g L^−1^), K_2_HPO_4_·3H_2_O (2.00 g L^−1^), ammonium citrate (2.00 g L^−1^), Tween 80 (1.00 g L^−1^), MgSO_4_·7H_2_O (0.20 g L^−1^) and MnSO_4_·4H_2_O (0.05 g L^−1^). For reactivation, bacteria were inoculated into Tryptone Peptone Yeast Extract (TPY) and De Man, Rogosa, and Sharpe (MRS) broths and incubated overnight at 37 °C under anaerobic conditions.

**Table 1:** Probiotic strains used in the present study.

Probiotic strains	Commercial name
*Lacticaseibacillus casei*	DSM 20011
*Lactobacillus acidophilus*	DSM 20079
*Bifidobacterium animalis* subsp. *lactis*	DSM 10140
*Bifidobacterium longum* subsp. *infantis*	DSM 20088
*Bifidobacterium bifidum*	DSM 20239

### 2.2 Growth conditions

Melon and watermelon seeds were obtained as powders from Sadullah Efendi (Batman, Turkiye). The composition of seeds is shown in Table 2.

**Table 2:** The composition of seeds.

Composition	Melon seed	Watermelon seed
Dry matter (g 100 g^−1^)	94.83	95.04
Ash (g 100 g^−1^)	8.07	7.26
Protein (g 100 g^−1^)	23.04	20.25
Fat (g 100 g^−1^)	35.00	28.43
Carbohydrate (g 100 g^−1^)	28.72	39.10
Glucose (g 100 g^−1^)	0.61	1.35
Galactose (g 100 g^−1^)	0.37	1.01
Fructose (g 100 g^−1^)	2.56	3.04
Sucrose (g 100 g^−1^)	0.75	0.07
Xylose (g 100 g^−1^)	0.04	0.01
Antioxidant capacity (DPPH µmol trolox g^−1^)	1.94	1.46
Total phenolic content (mg gallic acid g^−1^)	36.72	0.37

Glucose and inulin were eluted by using distilled water (10 % w/v) and filtered with a Millipore-Stericup-GP 0.45 μm [35]. No defatting process was applied to the seed powders prior to aqueous extraction. Seed powders were extracted with deionized water at a concentration of 10 % (w/v) using a modified procedure adapted from Sousa et al. [36]. Briefly, the powders were dispersed in deionized water and stirred continuously overnight at ambient temperature to facilitate the extraction of water-soluble components. Following extraction, the suspensions were centrifuged at 4,000 rpm for 25 min to remove insoluble material. The resulting supernatants were carefully recovered and subsequently sterilized by membrane filtration through a 0.45-µm pore-size filter (Millipore Stericup-GP). The resulting sterile, water-extractable seed fractions were used as test substrates in the subsequent fermentation experiments. To test the effect of seeds on bacterial metabolic activity, the basal carbohydrate-free medium, Tryptone Peptone Yeast Extract (TPY) broth for *Bifidobacterium* species, and a De Man Rogosa and Sharpe (MRS) broth for *Lactobacillus/Lacticaseibacillus* species were prepared. Both media were sterilized at 121 °C for 15 min. Filter-sterilized seed extracts were aseptically added to the respective carbohydrate-free basal media to achieve a concentration in the final fermentation medium equivalent to 2 % (w/v) of the initial seed material. This level was selected to standardize substrate loading across the experimental media, using the 2 % (w/v) glucose concentration commonly employed in standard commercial media as an experimental reference. Importantly, because the aqueous seed extracts represented complex water-extractable fractions containing carbohydrates together with other soluble seed constituents, the 2 % (w/v) seed-equivalent level did not represent a 2 % (w/v) pure carbohydrate concentration and was not intended to establish compositional equivalence with glucose or inulin. For *Bifidobacterium* species, carbohydrate-free TPY broth was used as the basal fermentation medium. Experimental media consisted of TPY without an added carbon source (negative control), TPY supplemented with glucose (2.00 % w/v; Merck, Germany), TPY supplemented with inulin (2.00 % w/v; Orafti^®^ HSI, Belgium) as the reference prebiotic substrate, and TPY supplemented with the aqueous melon or watermelon seed extract at a level corresponding to 2 % (w/v) initial seed material. The same experimental design was applied to *Lactobacillus/Lacticaseibacillus* species using carbohydrate-free MRS broth as the basal medium. Accordingly, MRS media contained no added carbon source, 2.00 % (w/v) glucose, 2.00 % (w/v) inulin, or the respective seed extract corresponding to 2 % (w/v) initial seed material. Each probiotic culture, following the second activation, was individually inoculated into the respective experimental medium at 2 % (v/v) and incubated anaerobically at 37 °C for 48 h using anaerobic incubation jars and the Anaerocult system. Each strain was evaluated independently in a monoculture-based *in vitro* fermentation model designed to characterize the direct fermentability of the water-extractable seed fractions by the selected probiotic strains. As simulated gastrointestinal digestion and fecal microbiota fermentation were not included in this experimental design, the resulting responses were interpreted as preliminary *in vitro* evidence of strain-specific substrate utilization rather than as direct evidence of gastrointestinal prebiotic efficacy.

### 2.3 Evaluation of bacterial metabolic activity

Bacterial metabolic activity during fermentation was monitored by measuring pH changes and optical density (OD_600_). The pH of the samples was measured at different incubation times (0, 12, 18, 24, 36 and 48 h) during the *in vitro* fermentation procedure using a pH meter (pH 315i/SET; WTW, Germany). All experimental procedures were performed in triplicate. The cell density of the samples was measured by monitoring the optical density at 600 nm (OD_600_) with a spectrophotometer (Shimadzu UV 1,800) at 0, 12, 24, 36, and 48 h of the *in vitro* fermentation procedure. The sterile TPY and MRS broth without bacteria were chosen as blanks for the absorbance measurements. Each experiment was repeated three times.

### 2.4 Prebiotic activity score (PAS) assay

The prebiotic activity score (PAS) was determined using the method reported by Huebner et al. [37], to compare the utilization of test substrates by probiotic strains with that of glucose and to evaluate this response relative to the substrate utilization of the reference enteric microorganism, *Escherichia coli*. TPY medium was used for *Bifidobacterium* species and MRS medium for *Lactobacillus/Lacticaseibacillus species*; the experimental media were prepared to contain 2.00 % (w/v) glucose, 2.00 % (w/v) inulin, or aqueous extracts of melon and watermelon seeds equivalent to 2 % (w/v) of the initial seed material in the final medium. The exact process was performed for *E. coli* using tryptic soy broth (TSB; Tryptone 17.00 g L^−1^; NaCl 5.00 g L^−1^; Bacto soytone 3.00 g L^−1^; glucose 2.50 g L^−1^; K_2_HPO_4_ 2.50 g L^−1^). Subsequently, 1 % of the inoculum of each strain was added to TSB and MRS media with test substrates. Strains were incubated at 37 °C for 24 h in anaerobic conditions. Samples were taken at 0 and 24 h of the *in vitro*-fermentation procedure, and the cell counts were evaluated using TPY agar for *Bifidobacterium* species, MRS agar for *Lactobacillus/Lacticaseibacillus* species, and Tryptic Soy agar for *E. coli*. Cell counts are expressed as log CFU mL^−1^. PAS was calculated according to Huebner et al. [37] by subtracting the ratio calculated for *E. coli* – derived from the change in probiotic cell count on the test substrate (0–24 h) relative to the corresponding change in a glucose medium – from the similarly calculated ratio for the probiotic. Positive PAS values indicate that the level of probiotic growth on the test substrate, normalized to glucose, exceeds the corresponding relative growth determined for *E. coli*. In this approach, *E. coli* served as the reference enteric microorganism to maintain comparability with the PAS methodology defined by Huebner et al. [37], and the resulting PAS values were interpreted as a comparative *in vitro* index quantifying the differences in relative utilization of the test substrates by the selected probiotic strains versus *E. coli*, rather than as a measure of general gut microbiota selectivity.

**Figure:**
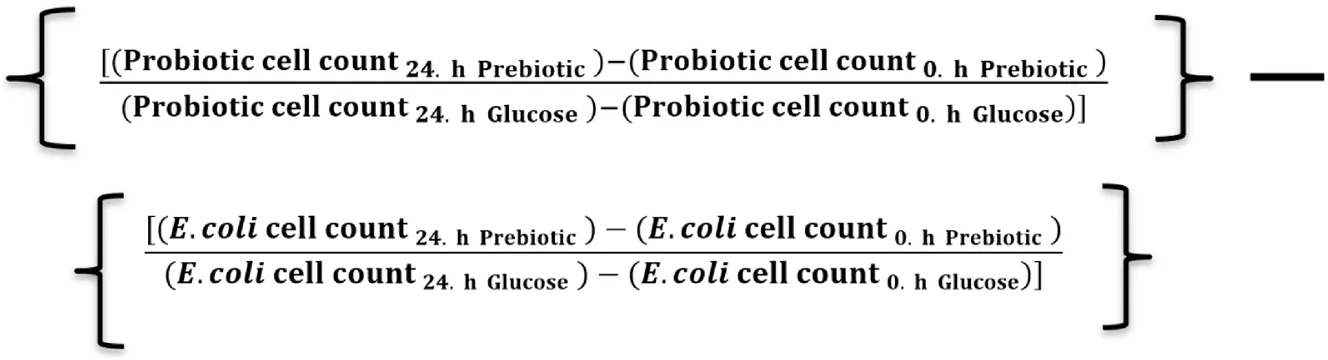


### 2.5 Sugar compound assay

Glucose, fructose, galactose, xylose, and sucrose were determined in samples collected at 0 and 48 h of *in vitro* fermentation using a Shimadzu Prominence high-performance liquid chromatography (HPLC) system (Shimadzu, Tokyo, Japan) equipped with a CBM-20A system controller, LC-20AT pump, SIL-20ACHT autosampler, CTO-10ASVp column oven, and RID-10A refractive index detector. Chromatographic separation was performed on an Inertsil NH_2_ column (250 × 4.6 mm, 5 µm) using methanol:water (80:20, v/v) as the mobile phase at a flow rate of 1.0 mL min^−1^ and a column temperature of 30 °C. Prior to analysis, liquid samples were filtered and subsequently injected into the HPLC system. Chromatographic data were acquired and processed using LC Solution software (Shimadzu). Individual sugars were identified by comparison with the corresponding analytical standards and quantified by external standard calibration using independently constructed calibration curves for each analyte. The calibration curves exhibited excellent linearity, with coefficients of determination (*R* ^2^) of 0.9991, 0.9996, 0.9999, 0.9999, and 0.9992 for xylose, fructose, glucose, galactose, and sucrose, respectively. The corresponding calibration curves are provided in the Supplementary material. The limit of detection (LOD) was 8.88 mg L^−1^ for fructose, glucose, and sucrose. Sugar concentrations were calculated using the respective calibration equations and expressed as mg L^−1^ [36].

### 2.6 Lactic acid and SCFA assays

Lactic acid and short-chain fatty acids (SCFAs; acetic, propionic, and butyric acids) were determined after 48 h of *in vitro* fermentation in media supplemented with glucose, inulin, melon seed extract, or watermelon seed extract, as well as in the negative control without an added carbon source. Lactic acid was quantified using a Shimadzu Prominence high-performance liquid chromatography system (Shimadzu, Japan) equipped with a CBM-20A system controller, LC-20AT pump, SIL-20ACHT autosampler, CTO-10ASVp column oven, and SPD-M20A diode-array detector (DAD). Chromatographic separation was performed on an Inertsil ODS-4 reversed-phase column (250 × 4.6 mm, 5 µm; GL Sciences, Japan). Ultrapure water adjusted to pH 3.0 with orthophosphoric acid was used as the mobile phase under isocratic conditions at a flow rate of 1.0 mL min^−1^. The injection volume was 20 µL, and detection was performed at 190 nm. Lactic acid was identified by matching its retention time with that of the corresponding analytical standard and quantified using an external standard calibration curve. Chromatographic data were acquired and processed using LC Solution software. The retention time (Rt) of lactic acid was 5.70 min, with a limit of detection (LOD) of 6.45 mg L^−1^ and a recovery of 93 %. The corresponding calibration curve is provided in the Supplementary material. Acetic, propionic, and butyric acids were determined using an Agilent 7890A gas chromatograph coupled to a 5975C mass selective detector and an Agilent 7697A Headspace Sampler (Agilent Technologies, USA). For analysis, 4 g/4 mL of each sample was transferred into a headspace vial. The vial oven, sampling needle, and transfer line temperatures were maintained at 85, 90, and 120 °C, respectively. Headspace equilibration, pressurization, injection, and withdrawal times were 5.0, 0.5, 0.08, and 0.5 min, respectively. Helium was used as the carrier gas at a system pressure of 25 psi. Chromatographic separation was achieved using a CP-Wax 52 CB capillary column (50 m × 0.25 mm i.d., 0.20 µm film thickness). The injector temperature was maintained at 180 °C. The oven temperature was initially held at 35 °C for 5 min, increased to 150 °C at 5 °C min^−1^, and maintained at 150 °C for an additional 5 min. Mass spectrometric detection was performed in electron ionization (EI) mode at 70 eV, with the ion source maintained at 250 °C and mass spectra acquired over an *m*/*z* range of 25–500. Acetic, propionic, and butyric acids were identified by comparison of their retention times with those of the corresponding analytical standards, with retention times of 24.5, 26.7, and 28.8 min, respectively. Quantification was performed using independently prepared external standard calibration curves for each analyte, all of which exhibited high linearity (*R* ^2^ = 0.999). The calibration curves are provided in the Supplementary material. The LOD values for acetic, propionic, and butyric acids were 1.46, 1.82, and 0.86 mg L^−1^, respectively. Analyte concentrations were calculated from the corresponding calibration equations [38].

### 2.7 Statistical analysis

Statistical analyses were performed using Minitab 17 (Minitab Inc., State College, PA, USA). Experimental data were evaluated using factorial analysis of variance (ANOVA) with substrate type, probiotic strain, and fermentation time as the main factors; in addition to the main effects, the interactions of “substrate type × probiotic strain”, “substrate type × fermentation time”, “probiotic strain × fermentation time”, and “substrate type × probiotic strain × fermentation time” were included in the model. Each experimental combination was evaluated using independent fermentations, and analyses at different time points were conducted in separate experimental tubes. For effects found to be significant by ANOVA, means were separated using Fisher’s least significant difference (LSD) multiple comparison test. Statistical significance was assessed at the *p* < 0.05 level, and effects at the *p* < 0.01 level were noted separately. Results are presented as mean ± standard deviation, and statistically different means are indicated by different letters. Principal component analysis (PCA) and hierarchical clustering with heatmap visualization were performed using SPSS Statistics (IBM, USA). The heat map for cluster analysis was generated in GraphPad Prism (GraphPad Software, Boston, USA).

## 3 Results

### 3.1 Bacterial metabolic activity

During fermentation, the growth kinetics of probiotic strains on different substrates were evaluated through changes in optical density (OD) and viable cell counts (Table 3). OD profiles demonstrated that fermentative growth exhibited different kinetics depending on both the substrate and the strain. For *L. casei*, the highest OD at 48 h was determined as 2.281 in glucose medium, while values of 1.451, 1.149, and 1.076 were obtained in inulin, melon seed, and watermelon seed, respectively. Similarly, for *L. acidophilus*, the highest OD at the end of fermentation was observed in glucose (2.069), followed by inulin (1.159), watermelon seed (0.969), and melon seed (0.920). In bifidobacteria, substrate-dependent OD variations exhibited a more heterogeneous pattern. For *B. lactis*, 48-h OD values were determined as 1.775, 1.295, 0.664, and 0.453 in glucose, inulin, melon seed, and watermelon seed, respectively, while in *B. infantis* the same values were 1.649, 0.738, 0.712, and 0.722; and in *B. bifidum* they were found as 1.343, 1.376, 1.003, and 0.905. This distribution revealed that the OD response in seed extracts, in particular, varied markedly across probiotic strains. Culture-based microbiological analyses corroborated the growth kinetics monitored by OD, revealing substrate-dependent differences in growth (Table 3). *L. casei* increased from an initial level of 6.62 log CFU mL^−1^ to 10.15 log CFU mL^−1^ at 48 h in glucose medium, while at the end of fermentation, values of 8.80, 8.69, and 8.95 log CFU mL^−1^ were obtained in inulin, melon seed, and watermelon seed, respectively. For *L. acidophilus*, while the cell count decreased to 4.00 log CFU mL^−1^ at 48 h in the negative control, it remained at levels of 8.84 and 8.58 log CFU mL^−1^ in melon seed and watermelon seed, respectively. Among the bifidobacteria, *B. lactis* reached 10.03 and 9.98 log CFU mL^−1^ at 48 h in inulin and watermelon seed, respectively. *B. infantis* reached a maximum level of 10.30 log CFU mL^−1^ at 24 h in watermelon seed and was determined as 9.08 log CFU mL^−1^ at 48 h, while 9.99 log CFU mL^−1^ was obtained in inulin at the end of fermentation. *B. bifidum* increased from an initial level of 7.03 log CFU mL^−1^ in watermelon seed to 9.67 log CFU mL^−1^ at 48 h. When OD and culture-based counts were evaluated together, it was notable that growth responses did not change in a synchronous manner across all substrate–strain combinations. One of the most prominent examples of this was the B*. lactis*–watermelon seed combination, where the OD was 0.453 at 48 h while the viable cell count reached 9.98 log CFU mL^−1^. The acidification kinetics accompanying microbial growth are shown in Figure 1. Although pH reduction depended on fermentation time in all probiotic strains, the magnitude of this change varied with substrate and strain. While the most pronounced pH decrease in *L. casei* and *L. acidophilus* was detected in glucose medium, inulin caused a more limited acidification; in melon and watermelon seed substrates, pH values were maintained at relatively high levels. A similar substrate differentiation was also observed for *B. lactis* and *B. bifidum*, with glucose and inulin producing more pronounced pH decreases than seed extracts. In contrast, *B. infantis* differentiated with a generally lower pH profile among the strains examined, and the differences among substrates became more pronounced in the later stages of fermentation. The temporal profiles in Figure 1 revealed that acidification continued throughout fermentation and that the magnitude of the response varied with substrate–strain interactions. The statistical basis of the growth and acidification patterns observed in Table 3 and Figure 1 was confirmed by the factorial analysis presented in Table 4. The main effects of substrate type, probiotic strain, and fermentation time on both pH and OD were found to be significant; all interactions of substrate × strain, substrate × time, strain × time, and substrate × strain × time also showed statistical significance for both variables (*p* < 0.01). In terms of substrate main effect, glucose produced the highest mean OD of 1.154 and the lowest mean pH value of 4.87; inulin followed with 0.812 OD and 5.43 pH. While the mean OD values in melon and watermelon seed media were 0.650 and 0.629, respectively, the pH values were in the same statistical group, at 5.88 and 5.87, respectively. The negative control showed the lowest OD of 0.411 and the highest pH mean of 6.03. When the main effect of probiotic strains was examined, the highest mean OD was determined in *L. casei* (0.883), followed by *B. bifidum* (0.788), *B. infantis* (0.760), *L. acidophilus* (0.644), and *B. lactis* (0.581). In contrast, the lowest overall pH was detected in *B. infantis* at 4.96; *B. bifidum* showed 5.34, *L. acidophilus* 5.91, while *L. casei* and *B. lactis* displayed mean pH values of 5.94. The effect of fermentation time revealed the temporal difference between growth and acidification kinetics more clearly. Mean OD increased from the initial level of 0.101–0.406 at 12 h and to 1.011 at 24 h, reached a maximum of 1.105 at 36 h, and decreased to 1.033 at 48 h. In contrast, the mean pH decreased continuously from 6.69 to 6.00, 5.21, 5.12, and 5.06 during the same time interval. Thus, although OD reached a maximum at 36 h followed by a limited decline, acidification continued until 48 h, revealing that cellular growth and medium acidification followed different temporal profiles. The culture-based counterpart of these growth dynamics was evaluated through viable cell counts in Table 5. The main effects of substrate type, probiotic strain, and fermentation time on viable cell density, as well as all binary and ternary interactions, were significant (*p* < 0.01). In terms of substrate means, glucose produced the highest cell density at 8.57 log CFU mL^−1^, followed by watermelon seed (8.21), inulin (8.11), melon seed (7.94), and negative control (7.35 log CFU mL^−1^). When the overall viable cell counts of probiotic strains were compared, the highest value was determined in *B. infantis* (8.54 log CFU mL^−1^), followed by *L. casei* (8.30), *B. bifidum* (8.12), *B. lactis* (7.94), and *L. acidophilus* (7.29 log CFU mL^−1^), respectively. When time-dependent changes in viable cells were evaluated alongside OD kinetics, the growth phases of fermentation became clearer. Overall viable cell density increased from the initial 6.87 log CFU mL^−1^ to 8.57 log CFU mL^−1^ at 24 h and reached 8.67 log CFU mL^−1^ at 48 h. While the initial value was significantly different from those at 24 and 48 h, the latter two time points were in the same statistical group. Accordingly, the majority of culture-based cell increase occurred within the first 24 h, with limited change in total cell density occurring between 24 and 48 h. In contrast, the increase in OD up to 36 h and the continued decrease in pH up to 48 h indicated that optical and metabolic changes persisted despite limited net cell proliferation during the later stages of fermentation. The significance of the substrate × strain × time interaction in all of OD, pH, and viable cell counts (*p* < 0.01) revealed that the fermentative response could not be explained by a single main factor and that the observed kinetics were determined by specific substrate–probiotic–time combinations.

**Table 3:** Optical density (OD) and changes in the microorganism counts (log CFU mL^−1^) in media supplemented with different substrates.

	OD					Log CFU/mL		
	0 h	12 h	24 h	36 h	48 h	0 h	24 h	48 h
***L. casei***								

Negative control	0.027 ± 0.002^cE^	0.252 ± 0.010^cD^	1.026 ± 0.000^dA^	0.932 ± 0.029^dB^	0.452 ± 0.001^eC^	6.62 ± 0.008^aB^	8.92 ± 0.494^aA^	8.70 ± 0.220^bA^
Glucose	0.030 ± 0.000^cE^	0.267 ± 0.003^bD^	1.900 ± 0.024^aC^	2.203 ± 0.013^aA^	2.281 ± 0.006^aA^	6.62 ± 0.191^aB^	9.57 ± 0.053^aA^	10.15 ± 0.681^aA^
Inulin	0.027 ± 0.004^cE^	0.228 ± 0.008^dD^	1.447 ± 0.016^bB^	1.318 ± 0.003^bC^	1.451 ± 0.002^bA^	6.60 ± 0.430^aB^	8.83 ± 0.018^aA^	8.80 ± 0.217^bA^
Melon seed	0.047 ± 0.003^bE^	0.324 ± 0.006^aD^	1.350 ± 0.002^cA^	1.266 ± 0.005^cB^	1.149 ± 0.005^cC^	6.76 ± 0.082^aB^	9.21 ± 0.647^aA^	8.69 ± 0.060^bA^
Watermelon seed	0.059 ± 0.001^aE^	0.322 ± 0.003^aD^	1.345 ± 0.025^cA^	1.294 ± 0.006^bcB^	1.076 ± 0.001^dC^	6.77 ± 0.038^aB^	9.27 ± 0.056^aA^	8.95 ± 0.072^bA^

***L. acidophilus***								

Negative control	0.006 ± 0.001^cE^	0.063 ± 0.003^eD^	0.365 ± 0.002^eA^	0.257 ± 0.000^eB^	0.230 ± 0.005^eC^	6.00 ± 0.000^bB^	6.91 ± 0.079^cA^	4.00 ± 0.000^dC^
Glucose	0.003 ± 0.000^cE^	0.145 ± 0.002^aD^	1.786 ± 0.019^aC^	1.970 ± 0.028^aB^	2.069 ± 0.002^aA^	7.28 ± 0.000^aC^	8.95 ± 0.234^aA^	8.39 ± 0.014^bcB^
Inulin	0.002 ± 0.000^cE^	0.074 ± 0.003^dD^	1.342 ± 0.030^bA^	1.270 ± 0.022^bB^	1.159 ± 0.006^bC^	5.88 ± 0.000^bC^	8.04 ± 0.185^bA^	7.32 ± 0.165^cB^
Melon seed	0.024 ± 0.005^bE^	0.105 ± 0.004^cD^	1.067 ± 0.080^cA^	0.860 ± 0.001^cC^	0.920 ± 0.001^dB^	6.34 ± 0.618^bB^	8.55 ± 0.069^abA^	8.84 ± 0.231^aA^
Watermelon seed	0.039 ± 0.004^aD^	0.122 ± 0.001^bC^	0.621 ± 0.016^dB^	0.623 ± 0.003^dB^	0.969 ± 0.018^cA^	5.88 ± 0.172^bB^	8.49 ± 0.381^abA^	8.58 ± 0.255^abA^

***B. lactis***								

Negative control	0.075 ± 0.015^aD^	0.067 ± 0.003^aD^	0.178 ± 0.018^eC^	0.911 ± 0.001^cA^	0.505 ± 0.040^dB^	5.25 ± 0.358^cC^	7.51 ± 0.000^bcB^	9.36 ± 0.003^cA^
Glucose	0.067 ± 0.003^bD^	0.058 ± 0.002^bD^	1.389 ± 0.015^aC^	1.649 ± 0.022^aB^	1.775 ± 0.018^aA^	9.36 ± 0.008^aA^	8.04 ± 0.052^bB^	9.42 ± 0.005^bcA^
Inulin	0.064 ± 0.002^bC^	0.060 ± 0.003^bC^	1.268 ± 0.013^bB^	1.458 ± 0.008^bA^	1.295 ± 0.051^bB^	5.70 ± 0.005^bcB^	9.24 ± 0.545^aA^	10.03 ± 0.29^aA^
Melon seed	0.050 ± 0.003^dD^	0.020 ± 0.000^dD^	0.309 ± 0.000^dC^	0.742 ± 0.025^dA^	0.664 ± 0.016^cB^	5.69 ± 0.438^bcC^	7.45 ± 0.184^bcB^	8.70 ± 0.007^dA^
Watermelon seed	0.057 ± 0.005^cC^	0.046 ± 0.003^cC^	0.474 ± 0.013^cB^	0.888 ± 0.037^cA^	0.453 ± 0.003^dB^	6.17 ± 0.407^bB^	7.23 ± 0.097^cB^	9.98 ± 0.443^abA^

***B. infantis***								

Negative control	0.290 ± 0.007^cD^	0.546 ± 0.009^dA^	0.550 ± 0.014^aA^	0.461 ± 0.009^eB^	0.369 ± 0.020^cC^	7.74 ± 0.064^abB^	8.44 ± 0.020^cA^	7.52 ± 0.252^cB^
Glucose	0.283 ± 0.004^dE^	1.141 ± 0.005^aD^	1.567 ± 0.012^aC^	1.785 ± 0.003^aA^	1.649 ± 0.014^aB^	7.82 ± 0.115^aB^	8.80 ± 0.068^cA^	8.96 ± 0.219^bA^
Inulin	0.305 ± 0.002^cC^	0.837 ± 0.007^bA^	0.812 ± 0.004^bA^	0.812 ± 0.006^cA^	0.738 ± 0.028^bB^	7.74 ± 0.152^abC^	8.70 ± 0.431^cB^	9.99 ± 0.030^aA^
Melon seed	0.349 ± 0.000^bD^	0.792 ± 0.013^cA^	0.757 ± 0.002^cAB^	0.734 ± 0.025^dBC^	0.712 ± 0.008^bC^	7.58 ± 0.032^abC^	9.64 ± 0.189^bA^	8.34 ± 0.000^bcB^
Watermelon seed	0.362 ± 0.001^aE^	0.787 ± 0.012^cB^	0.754 ± 0.003^cC^	0.896 ± 0.009^bA^	0.722 ± 0.008^bD^	7.51 ± 0.165^bB^	10.30 ± 0.109^aA^	9.08 ± 0.659^bA^

***B. bifidum***								

Negative control	0.056 ± 0.004^cE^	0.654 ± 0.005^cC^	0.781 ± 0.032^dA^	0.691 ± 0.001^eB^	0.542 ± 0.004^eD^	7.20 ± 0.279^abC^	8.38 ± 0.236^bA^	7.82 ± 0.062^dB^
Glucose	0.044 ± 0.002^dD^	0.827 ± 0.002^aC^	1.247 ± 0.019^aB^	1.368 ± 0.052^aA^	1.343 ± 0.023^bA^	7.39 ± 0.393^abB^	8.79 ± 0.094^aA^	9.06 ± 0.476^bA^
Inulin	0.058 ± 0.004^cE^	0.761 ± 0.006^bD^	0.963 ± 0.011^cC^	1.168 ± 0.022^bB^	1.376 ± 0.011^aA^	7.67 ± 0.206^aB^	8.73 ± 0.181^aA^	8.39 ± 0.088^cA^
Melon seed	0.104 ± 0.003^aD^	0.827 ± 0.011^aC^	1.007 ± 0.013^bB^	1.076 ± 0.004^cA^	1.003 ± 0.006^cBC^	7.25 ± 0.065^abB^	8.02 ± 0.032^cA^	8.06 ± 0.024^cdA^
Watermelon seed	0.098 ± 0.001^bE^	0.828 ± 0.005^aD^	0.979 ± 0.002^bcB^	0.994 ± 0.007^dA^	0.905 ± 0.004^cC^	7.03 ± 0.040^bC^	8.30 ± 0.157^bcB^	9.67 ± 0.053^aA^

Values are expressed as mean ± standard deviation. ^a,b^lowercase letters indicate statistically significant differences (*p* < 0.01) between the substrates within the same fermentation time. ^A,B^uppercase letters represent statistically significant differences (*p* < 0.01) across fermentation times within the same substrate.

**Figure 1:**
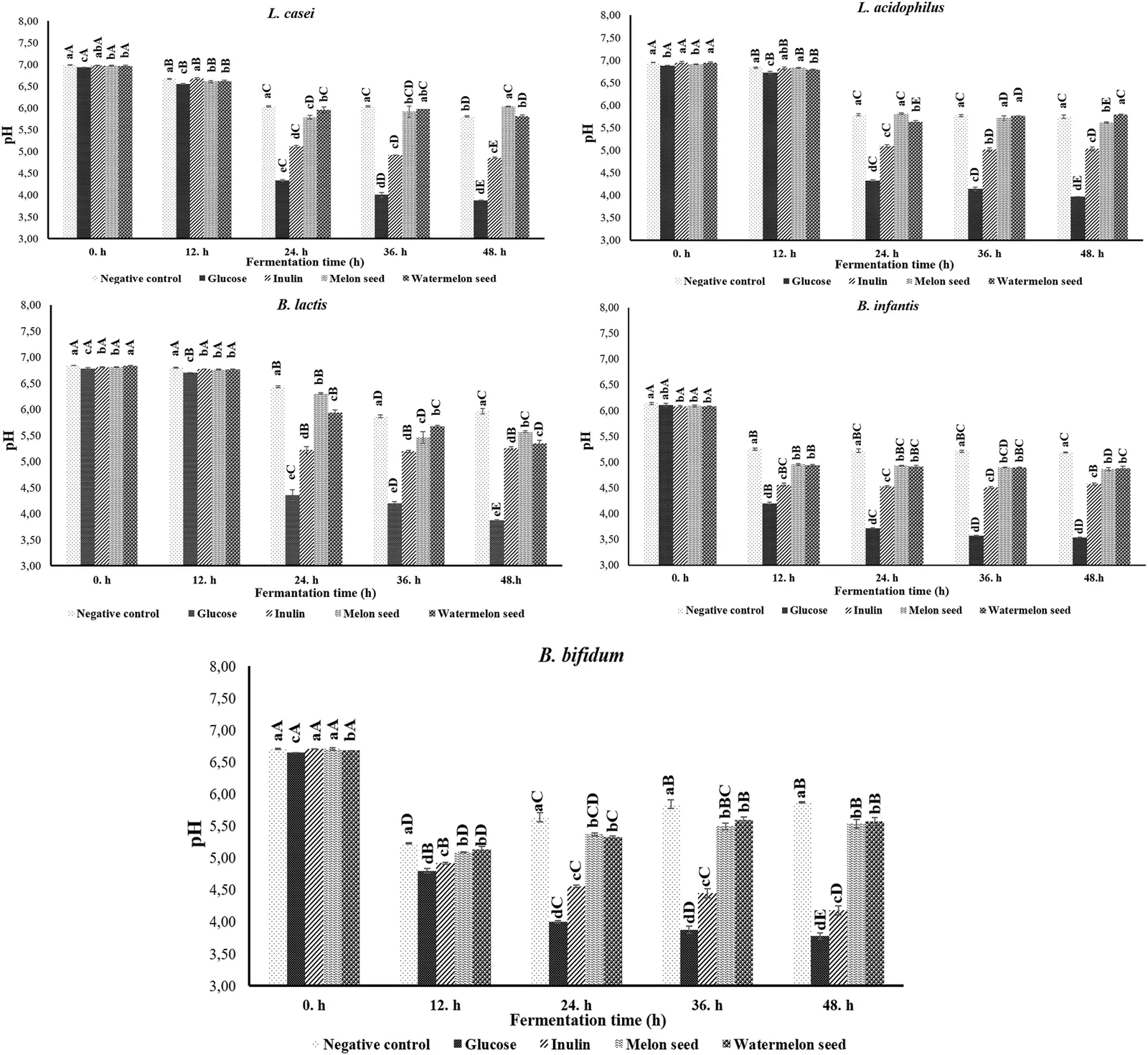
pH values of tested probiotic strains during fermentation. ^a,b^Lowercase letters indicate statistically significant differences (*p* < 0.01) between the substrates ^A,B^uppercase letters represent statistically significant differences (*p* < 0.01) across fermentation times.

**Table 4:** Variance analysis results of pH and OD values based on substrate types, probiotic strain, fermentation times, and the interaction among them.

Substrate types	*N*	pH	OD
Negative control	75	6.03^a^	0.411^e^
Glucose	75	4.87^d^	1.154^a^
Inulin	75	5.43^c^	0.812^b^
Melon seed	75	5.88^b^	0.650^c^
Watermelon seed	75	5.87^b^	0.629^d^

**Probiotic strains**			

*L. casei*	75	5.94^a^	0.883^a^
*L. acidophilus*	75	5.91^b^	0.644^d^
*B. lactis*	75	5.94^a^	0.581^e^
*B. infantis*	75	4.96^d^	0.760^c^
*B. bifidum*	75	5.34^c^	0.788^b^

**Fermentation time (h)**			

0	75	6.69^a^	0.101^e^
12	75	6.00^b^	0.406^d^
24	75	5.21^c^	1.011^c^
36	75	5.12^d^	1.105^a^
48	75	5.06^e^	1.033^b^

**ANOVA**			

Substrate type		**	**
Probiotic strain		**	**
Fermentation time		**	**
Substrate type × probiotic strain		**	**
Substrate type × fermentation time		**	**
Probiotic strain × fermentation time		**	**
Substrate type × probiotic strain × fermentation time		**	**

**Means with different letters are significantly different from each other within the same column; *p < 0.01; p < 0.05. N indicates the number of observations.

**Table 5:** Variance analysis results of microorganisms count based on substrate types, probiotic strain, fermentation times, and the interaction among them.

Substrate types	*N*	Log CFU/mL
Negative control	30	7.35^d^
Glucose	30	8.57^a^
Inulin	30	8.11^bc^
Melon seed	30	7.94^c^
Watermelon seed	30	8.21^b^

**Probiotic strains**		

*L. casei*	30	8.30^b^
*L. acidophilus*	30	7.29^d^
*B. lactis*	30	7.94^c^
*B. infantis*	30	8.54^a^
*B. bifidum*	30	8.12^c^

**Fermentation time (h)**		

0	50	6.87^b^
24	50	8.57^a^
48	50	8.67^a^

**ANOVA**		

Substrate type		**
Probiotic strain		**
Fermentation time		**
Substrate type × probiotic strain		**
Substrate type × fermentation time		**
Probiotic strain × fermentation time		**
Substrate type × probiotic strain × fermentation time		**

**Means with different letters are significantly different from each other within the same column; *p < 0.01; p < 0.05. N indicates the number of observations.

### 3.2 Prebiotic activity score (PAS)

The prebiotic activity scores (PAS) of melon and watermelon seed extracts on probiotic strains are presented in Figure 2. The PAS profiles demonstrated that the substrates did not produce a homogeneous response in probiotic microorganisms. While positive PAS values were obtained for *L. casei*, *L. acidophilus*, and *B. infantis* in melon seed, watermelon seed was characterized by more pronounced positive responses for *B. lactis* and *B. bifidum*. In contrast, a negative PAS was determined for the watermelon seed–*L. casei* combination. When this result was evaluated together with viable cell counts, it was observed that the negative PAS value did not indicate the absence of microbial growth. Indeed, *L. casei* count increased from 6.77 log CFU mL^−1^ to 8.95 log CFU mL^−1^ at 48 h in watermelon seed medium; however, the same strain reached 10.15 log CFU mL^−1^ in glucose medium. Thus, the PAS distribution revealed that the relative response to seed extracts varied depending on the probiotic strain.

**Figure 2:**
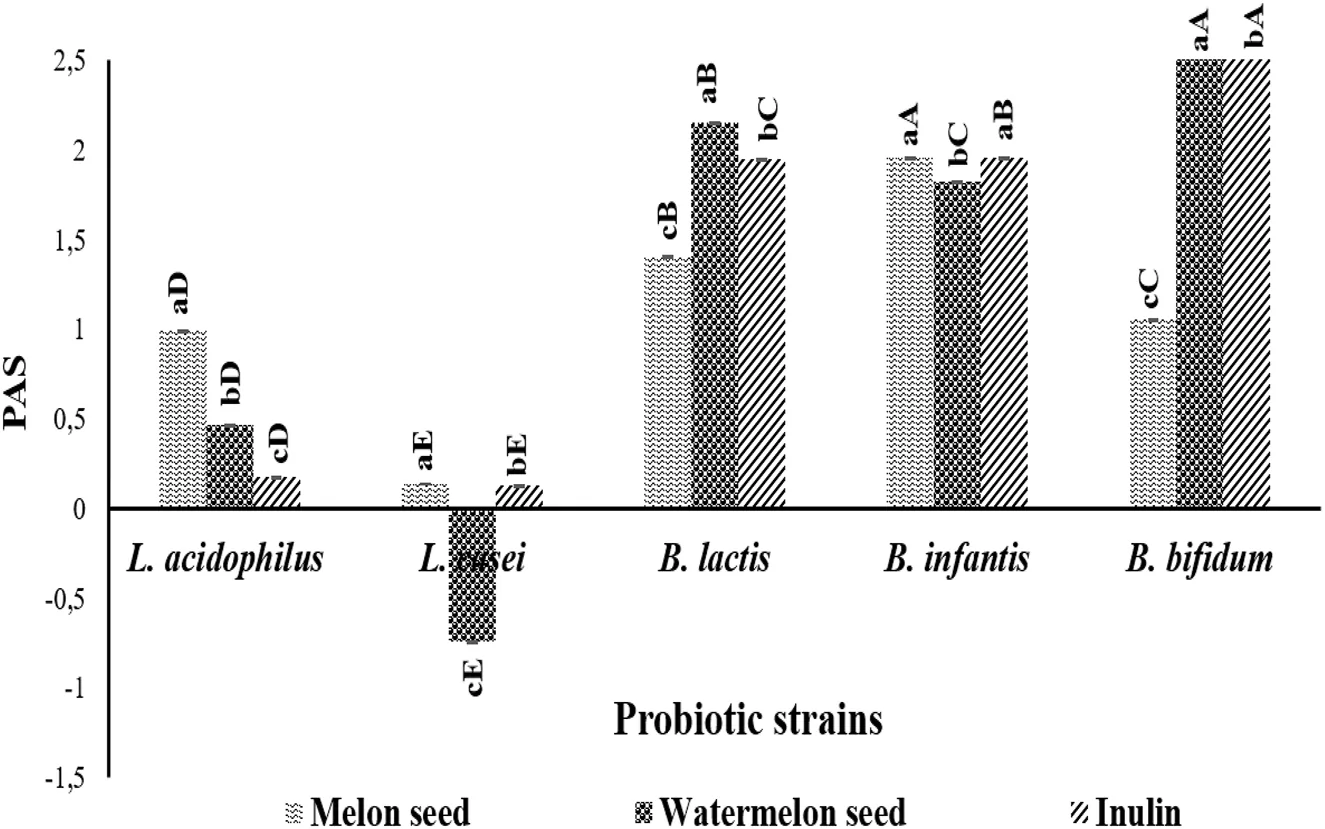
PAS values obtained from melon and watermelon seeds of different probiotic strains. ^a,b^Lowercase letters indicate statistically significant differences (*p* < 0.01) between the substrates. ^A,B^Uppercase letters represent statistically significant differences (*p* < 0.01) across probiotic strains.

### 3.3 Sugar compounds

The distribution of glucose, fructose, galactose, xylose, and sucrose concentrations at 0 and 48 h in inulin, melon seed, and watermelon seed media is shown in Figure 3. The heat map revealed that the initial sugar composition varied by substrate and probiotic strain, and that this profile changed significantly by 48 h of fermentation. Initially, the highest glucose concentration was determined in the inulin–*L. acidophilus* combination (8.70 g L^−1^), galactose in melon seed–*L. acidophilus* (21.78 g L^−1^), fructose in watermelon seed–*L. acidophilus* (21.19 g L^−1^), sucrose in inulin–*L. acidophilus* (13.43 g L^−1^), and xylose in inulin–*B. bifidum* (3.46 g L^−1^) combinations. At the end of fermentation, the highest glucose and galactose concentrations were detected in the melon seed–*B. bifidum* combination at 2.24 and 8.74 g L^−1^, respectively. The highest fructose was 3.89 g L^−1^ in the watermelon seed–*B. infantis* combination, while the highest values for sucrose and xylose at 48 h were determined in the inulin–*B. lactis* combination at 8.52 and 1.59 g L^−1^, respectively. Principal component analysis in Figure 3 demonstrated that this variability in the sugar profile formed distinct multivariate structures by substrate. The first two principal components explained 87.2 % of the total variance in inulin medium (PC1: 66.6 %; PC2: 20.6 %), 63.7 % in melon seed (PC1: 34.0 %; PC2: 29.7 %), and 80.1 % in watermelon seed (PC1: 59.5 %; PC2: 20.6 %). The positional changes of pre- and post-fermentation samples in the principal component space indicated that the sugar profile was restructured during the 48-h fermentation process. Analysis of variance for sugar components showed that substrate type, probiotic strain, and fermentation time had significant main effects on all five sugars studied, and all binary and tertiary interactions were also significant (*p* < 0.01; Table 6). In terms of substrate main effect, inulin had the highest means for glucose (3.48 g L^−1^), fructose (4.08 g L^−1^), xylose (1.44 g L^−1^), and sucrose (7.75 g L^−1^), while melon seed distinguished itself from other substrates with 6.95 g L^−1^ for galactose. In watermelon seed, the means for glucose, fructose, galactose, xylose, and sucrose were determined as 2.69, 3.97, 4.80, 1.20, and 4.09 g L^−1^, respectively. Regarding probiotic strain main effect, the highest mean glucose was detected in *B. bifidum* (3.46 g L^−1^), fructose and xylose in *L. acidophilus* (5.82 and 1.61 g L^−1^, respectively), galactose in *L. casei* (7.14 g L^−1^), and sucrose in *B. lactis* (5.76 g L^−1^). Fermentation time caused a significant reduction in all sugars; between 0 and 48 h, glucose decreased from 4.33 to 1.05 g L^−1^, fructose from 4.29 to 2.20 g L^−1^, galactose from 6.53 to 2.91 g L^−1^, xylose from 1.57 to 0.82 g L^−1^, and sucrose from 6.49 to 4.17 g L^−1^ (*p* < 0.01).

**Figure 3:**
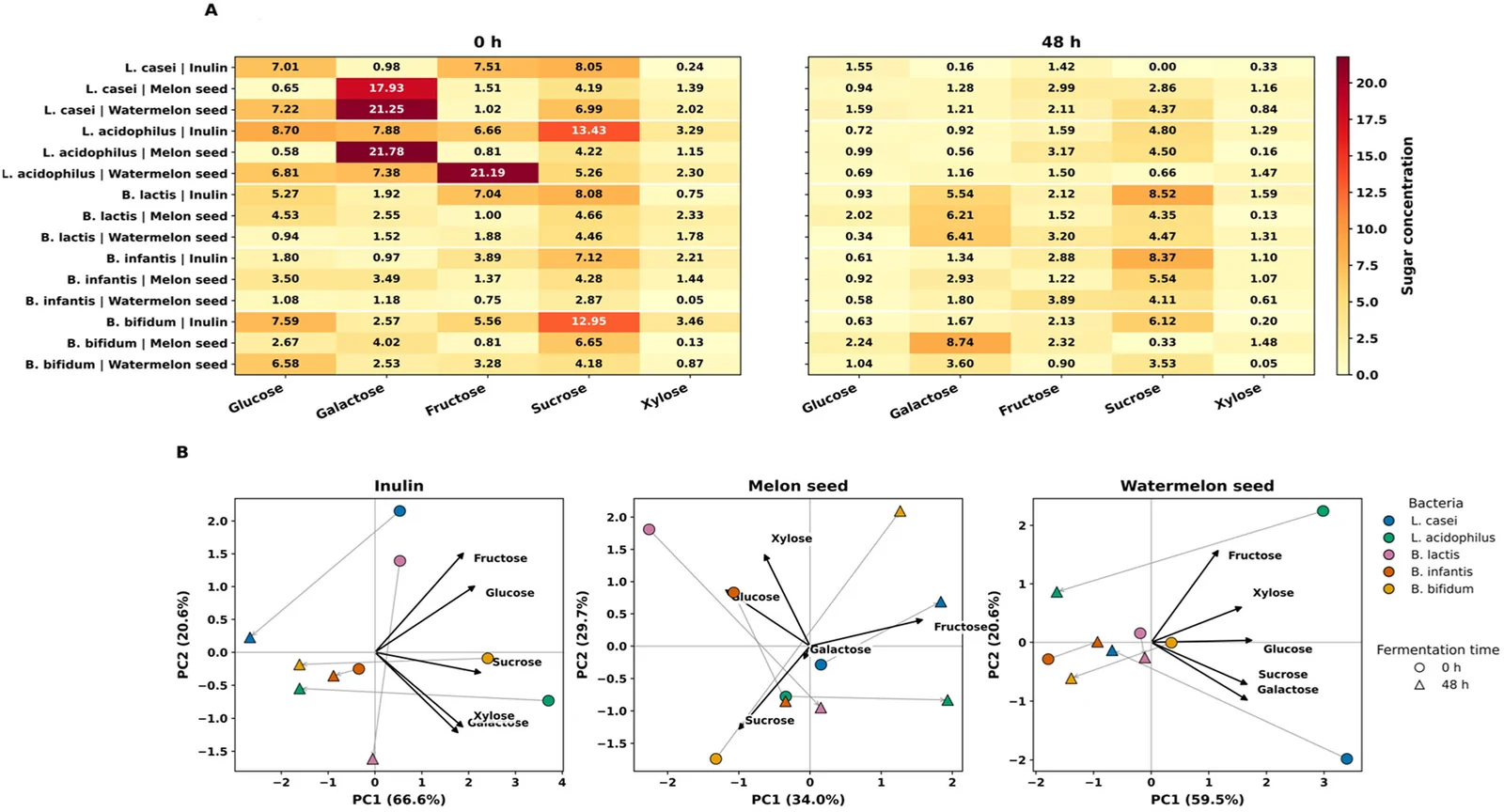
Sugar compounds determined at 0 h and 48 h of fermentation.

**Table 6:** Variance analysis results of sugar components based on substrate types, probiotic strain, fermentation times, and the interaction among them.

Substrate types	*N*	Glucose (g L^−1^)	Fructose (g L^−1^)	Galactose (g L^−1^)	Xylose (g L^−1^)	Sucrose (g L^−1^)
Inulin	20	3.48^a^	4.08^a^	2.41^c^	1.44^a^	7.75^a^
Melon seed	20	1.90^c^	1.67^c^	6.95^a^	0.94^c^	4.16^b^
Watermelon seed	20	2.69^b^	3.97^b^	4.80^b^	1.20^b^	4.09^c^

**Probiotic strains**						

*L. casei*	12	3.16^b^	2.76^c^	7.14^a^	1.09^c^	4.41^e^
*L. acidophilus*	12	3.08^c^	5.82^a^	6.61^b^	1.61^a^	5.48^c^
*B. lactis*	12	2.34^d^	2.79^b^	4.04^c^	1.33^b^	5.76^a^
*B. infantis*	12	1.42^e^	2.33^e^	1.95^e^	0.91^e^	5.38^d^
*B. bifidum*	12	3.46^a^	2.50^d^	3.86^d^	1.03^d^	5.63^b^

**Fermentation time (h)**						

1 h	30	4.33^a^	4.29^a^	6.53^a^	1.57^a^	6.49^a^
48 h	30	1.05^b^	2.20^b^	2.91^b^	0.82^b^	4.17^b^

**ANOVA**						

Substrate type		**	**	**	**	**
Probiotic strain		**	**	**	**	**
Fermentation time		**	**	**	**	**
Substrate type × probiotic strain		**	**	**	**	**
Substrate type × fermentation time		**	**	**	**	**
Probiotic strain × fermentation time		**	**	**	**	**
Substrate type × probiotic strain × fermentation time		**	**	**	**	**

**Means with different letters are significantly different from each other within the same column; *p < 0.01; p < 0.05. N indicates the number of observations.

### 3.4 Lactic acid and SCFA generation

The concentrations of lactic acid, acetic, propionic, and butyric acids, as well as total SCFA values formed at the end of fermentation, are presented in Table 7. Substrate type, probiotic strain, and substrate × strain interaction showed significant effects on all metabolites examined (*p* < 0.01). Among the substrates, the highest lactic acid concentration was determined in inulin at 576.40 ppm, followed by watermelon seed (524.49 ppm), negative control (371.77 ppm), melon seed (276.33 ppm), and glucose (203.13 ppm). In contrast, the highest values for acetic acid, butyric acid, and total SCFA were detected in glucose medium at 27.70, 0.80, and 29.73 ppm, respectively. Propionic acid reached its maximum level in inulin at 1.57 ppm. Watermelon seed showed the lowest averages for acetic acid (0.07 ppm), butyric acid (0.22 ppm), and total SCFA (0.85 ppm). Total SCFA in melon seed was determined as 1.36 ppm. Regarding the main effect of strains, *L. casei* had the highest average lactic acid (543.57 ppm) and butyric acid (0.61 ppm), while *L. acidophilus* had the highest values for acetic acid (18.06 ppm), propionic acid (1.84 ppm), and total SCFA (20.47 ppm). *B. lactis* showed the lowest lactic acid concentration at 151.60 ppm. *B. bifidum* had the lowest strain averages in terms of acetic acid (0.26 ppm), propionic acid (0.40 ppm), and total SCFA (0.97 ppm). In the negative control medium, 371.77 ppm lactic acid and 1.63 ppm total SCFA were determined. Accordingly, while total SCFA concentrations in melon (1.36 ppm) and watermelon seed (0.85 ppm) media remained lower than the negative control, the lactic acid concentration in watermelon seed (524.49 ppm) was higher than the negative control.

**Table 7:** Variance analysis results of lactic acid and SCFA values based on substrate type, probiotic strain, and the interaction between them after 48-h fermentation.

Substrate types	*N*	Lactic acid (ppm)	Acetic acid (ppm)	Propionic acid (ppm)	Butyric acid (ppm)	Total SCFA (ppm)
Negative control	10	371.77^c^	0.83^b^	0.36^e^	0.44^c^	1.63^c^
Glucose	10	203.13^e^	27.70^a^	1.22^b^	0.80^a^	29.73^a^
Inulin	10	576.40^a^	0.36^d^	1.57^a^	0.48^b^	2.38^b^
Melon seed	10	276.33^d^	0.37^c^	0.66^c^	0.34^d^	1.36^d^
Watermelon seed	10	524.49^b^	0.07^e^	0.56^d^	0.22^e^	0.85^e^

**Probiotic strains**						

*L. casei*	10	543.57^a^	6.15^b^	0.60^d^	0.61^a^	7.36^b^
*L. acidophilus*	10	447.79^b^	18.06^a^	1.84^a^	0.57^b^	20.47^a^
*B. lactis*	10	151.60^e^	4.27^c^	0.80^b^	0.49^c^	5.56^c^
*B. infantis*	10	425.82^c^	0.55^d^	0.73^c^	0.32^d^	1.59^d^
*B. bifidum*	10	383.33^d^	0.26^e^	0.40^e^	0.31^d^	0.97^e^

**ANOVA**						

Substrate type		**	**	**	**	**
Probiotic strain		**	**	**	**	**
Substrate type x probiotic strain		**	**	**	**	**

**Means with different letters are significantly different from each other within the same column; *p < 0.01; p < 0.05. N indicates the number of observations.

### 3.5 Integrated multivariate fermentation response

To enable a holistic comparison of microbial growth, substrate selectivity, sugar utilization, and fermentation metabolites independent of different measurement scales, variables were standardized to z-scores, and the resulting response matrix is presented in Figure 4. Positive z-scores (red) indicate responses above the overall mean, while negative z-scores (blue) indicate responses below the overall mean. The analysis jointly evaluated microbial growth indicators (Δlog CFU mL^−1^ and ΔOD_600_), PAS, sugar utilization (consumption of galactose, xylose, sucrose, glucose, and fructose), and fermentation metabolites (lactic, propionic, acetic, and butyric acids, as well as total SCFA). The standardized response matrix revealed that increases in cellular proliferation and optical density do not represent the same fermentative response. The standardized multivariate profile presented in Figure 4 demonstrated that the fermentative responses of probiotic strains to substrates cannot be explained through a single growth or metabolic indicator. Although the highest Δlog CFU mL^−1^ response was determined in the inulin–*B. lactis* combination (*z* = 2.1), a similarly strong positive response in terms of ΔOD_600_ was not observed in the same combination; high ΔOD_600_ z-scores were concentrated in different strain–substrate pairings. This divergence demonstrated that viable cell increase and optical density-based biomass change exhibit different response patterns. Similarly, the PAS profile differed from microbial growth indicators; high PAS values did not show a systematic association with the strongest responses in Δlog CFU mL^−1^ and ΔOD_600_. This finding reveals that substrate utilization capacity and selective prebiotic efficacy represent distinct dimensions of biological response. When carbohydrate utilization profiles were examined, it was determined that high z-scores for glucose, fructose, galactose, xylose, and sucrose consumption did not cluster simultaneously in the same strain–substrate combinations. The heterogeneous distribution in monosaccharide and disaccharide utilization demonstrated that carbohydrate metabolism is distinctly strain- and substrate-dependent. Furthermore, the absence of a common high-consumption profile across all sugars in combinations exhibiting high cellular growth or PAS response indicated that microbial proliferation and prebiotic selectivity do not directly parallel total carbohydrate-utilization capacity. Fermentation metabolites similarly exhibited a differentiated distribution. Standardized responses for lactic, acetic, propionic, and butyric acids, along with total SCFA, did not concentrate together in the same strain–substrate combinations; notably, high lactic acid formation did not systematically overlap with high total SCFA or individual SCFA production. This situation demonstrates that lactic acid production and SCFA formation represent different endpoints of fermentative metabolism.

**Figure 4:**
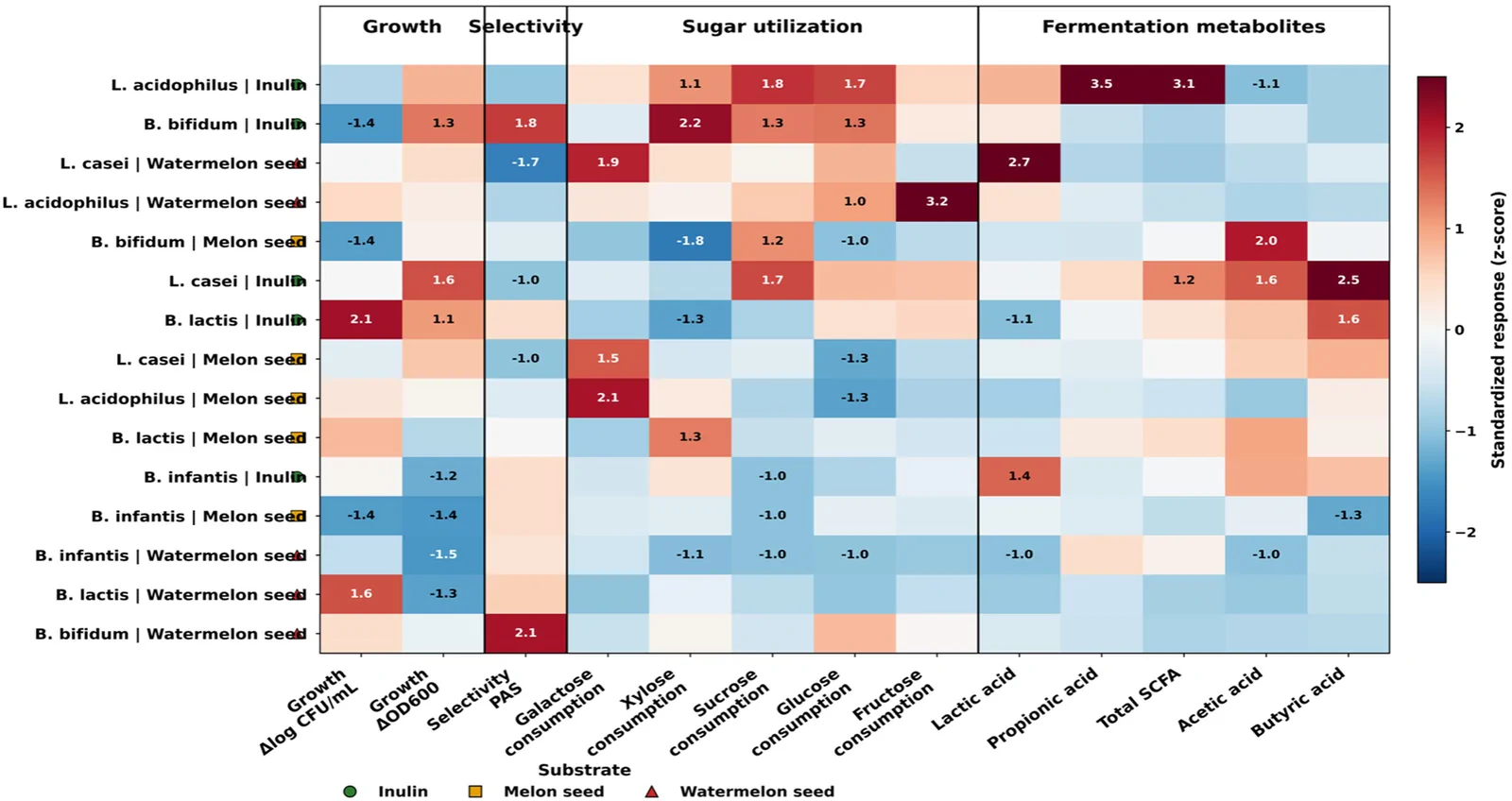
Integrated heatmap of standardized growth, substrate selectivity, sugar utilization, and fermentation metabolite responses of probiotic strains to different substrates.

## 4 Discussion

Prebiotic potential requires a holistic evaluation of the substrate’s selective fermentability and the accompanying metabolic responses, rather than merely microbial proliferation [6], 37], 42]. In this study, the differentiation among OD_600_, culture-based viable cell counts, and acidification kinetics revealed that the utilization of melon and watermelon seed fractions by probiotics depends on substrate–strain interactions. In particular, the lack of correspondence between OD_600_ and viable cell density in some combinations demonstrates the limitation of absorbance in representing cellular proliferation in complex plant matrices. While Swaroopa et al. [18] reported that seed-derived fractions could support probiotic proliferation, Mandalari et al. [17] and Pérez-Juárez et al. [34] showed that the utilization of plant substrates by bifidobacteria and lactobacilli can vary according to microorganism–substrate pairing. Similar differentiations reported by Tan et al. [19] and Akter et al. [21] also support that the fermentative functionality of plant by-products is associated not only with carbohydrate content but also with the quality of accessible components and the metabolic repertoire of the microorganism. Furthermore, because the cellular activity observed in the negative control indicates that the basal medium is not completely metabolically inert, the effect of seed fractions should be interpreted in terms of integrated proliferative and fermentative responses relative to the control system rather than as absolute changes in cell density or pH.

Unlike absolute cell density, PAS allows for the differentiation of fermentability and prebiotic selectivity, as it quantifies the relative advantage provided by the candidate substrate to the target probiotic compared to glucose and enteric reference microorganisms [37]. In the current study, the differentiation of positive PAS values by probiotic–seed pairing suggests that seed fractions exert a strain-specific selective effect rather than a general prebiotic property. Pallavi et al. [14] reported that even different carbohydrate fractions of the same plant material can generate different PAS values; Zhang et al. [15] reported that the selectivity of pectin-derived oligosaccharides differed on *L. paracasei* and *B. bifidum*. Consistent with the substrate structure–microbial metabolic capacity relationship defined by Rastall and Gibson [42], the current PAS distribution also supports the view that prebiotic selectivity depends on the compatibility between accessible substrate characteristics and bacterial metabolic machinery rather than on botanical source. However, since seed extracts contain complex water-extractable fractions, it is not appropriate to attribute the determined selectivity solely to carbohydrate components.

The restructuring of the sugar profile during fermentation indicates that microbial utilization of kernel fractions is governed by a more complex metabolic process than the initial free carbohydrate concentration alone. The molecular size of carbohydrates, degree of polymerization, and glycosidic bond characteristics are fundamental determinants of microbial accessibility and fermentative selectivity [43]. The differentiation of sugar changes according to strain and substrate, and the emergence of substrate-specific variation patterns in PCA, suggests that carbohydrate transformation cannot be explained solely by taxonomic identity. Pokusaeva et al. [44] associated the metabolic diversity in the capacity of *Bifidobacterium* species to utilize different carbohydrates with substrate-specific transport systems and the enzymatic activity of glycosyl hydrolases. Therefore, the changes observed at the end of fermentation reflect not only sugar consumption but also the net carbohydrate transformation, which is a composite of complex carbohydrate hydrolysis, release of free sugars, and simultaneous microbial utilization.

The lack of parallel distribution between lactic acid and SCFA profiles and cellular proliferation indicators demonstrates that carbon flux derived from the substrate can be directed differently toward biomass formation and fermentative end-product synthesis. In particular, the absence of a simultaneous increase in lactic acid formation and total SCFA levels for the same substrates, and the differentiation of metabolite distributions among strains, reveal that the fermentative phenotype cannot be explained solely by cell density or taxonomic characteristics. Pérez-Juárez et al. [34], Kandil and Yilmaz-Ersan [45], Usta-Gorgun and Yilmaz-Ersan [46], and Omak and Yilmaz-Ersan [47] reported that different plant-based or carbohydrate sources can redirect SCFA metabolism depending on the microorganism; Eroglu and Ozcan [48] reported that substrate composition can affect probiotic viability and SCFA formation at different levels. The detection of fermentative metabolites in the negative control and the possibility that total SCFA levels in kernel media may remain below the control further demonstrate that not all metabolites can be directly attributed to kernel carbohydrates and that these substrates should not be assigned a general SCFA-enhancing property. These findings support the view that cellular proliferation and metabolic end-product formation are related but not directly equivalent fermentative processes.

The integration of microbial proliferation, PAS, carbohydrate conversion, and fermentative metabolites in Figure 4 has revealed that the functionality of melon and watermelon seeds cannot be defined by a single response variable. The fact that high cellular proliferation or selectivity does not systematically overlap with an equivalent response in carbohydrate conversion and metabolite formation suggests that each probiotic–substrate pairing generates a unique fermentative phenotype. Gómez-García et al. [41] and Pérez-Juárez et al. [34] reported that microbial utilization of melon and watermelon-derived by-products varies according to matrix and microorganism characteristics; while Rolim et al. [49] indicated that melon seed possesses fermentative substrate potential for *B. lactis*. The relationship between chemical structure, biological accessibility, and microbial metabolism, as emphasized by Ghazaly et al. [50], also provides a biological framework for this matrix-dependent behavior. However, because the current monoculture *in vitro* system does not permit direct inference about gastrointestinal resistance, complex microbiota interactions, or host responses, the findings should be evaluated in the context of strain-specific *in vitro* fermentability and selectivity rather than as definitive evidence of prebiotic efficacy. Within this framework, the functional potential of melon and watermelon seeds can be defined as a selective fermentative characteristic shaped by the specific compatibility between the substrate’s profile of accessible components and the target probiotic’s metabolic capacity, rather than as a general superiority applicable to all probiotics.

## 5 Conclusions

This study has demonstrated that the fermentative potential of water-extractable fractions from melon and watermelon seeds is determined by strain-substrate specificity among microbial growth, selective substrate utilization, carbohydrate conversion, and metabolite formation. The divergence in carbohydrate conversion, lactic acid, and short-chain fatty acid profiles has shown that microbial growth does not directly correlate with metabolic end-product formation and that the functionality of seed fractions cannot be defined by a single fermentative indicator. These findings position melon and watermelon seeds as potential prebiotic substrates exhibiting strain-specific selective fermentability, rather than as proven prebiotics. Thus, a scientific foundation is provided for the transformation of *Cucurbitaceae* seeds into microbiota-targeted functional components within the framework of circular bioeconomy. However, further research, including simulated gastrointestinal digestion, complex fecal microbiota fermentation, and *in vivo* studies, is needed to validate prebiotic functionality.

## Supplementary Material

Supplementary Material
