# Supplementary material for: In vitro characterization of the candidate prebiotic potential of melon and watermelon seeds: strain-specific fermentation and targeted metabolic profiling
Source: Open Life Sci. 2026 Oct 12;21(1):20251386. doi: 10.1515/biol-2025-1386 (PMC13656051; doi:10.1515/biol-2025-1386)
Supplement: Supplementary file 1 [file j_biol-2025-1386_suppl_001.docx]

|  |  |
| --- | --- |
| 30000 | 548882 |
| 15000 | 263439 |
| 7500 | 135076 |
| 3750 | 59830 |
| 1875 | 19210 |
| 937.5 | 18410 |

**Figure 1:** Calibration curve for the xylose standard

|  |  |
| --- | --- |
| 28000 | 620450 |
| 14000 | 301747 |
| 7000 | 157997 |
| 3500 | 78002 |
| 1750 | 33014 |
| 875 | 14269 |

**Figure 2:** Calibration curve for the fructose standard

|  |  |
| --- | --- |
| 30000 | 471535 |
| 15000 | 236635 |
| 7500 | 114801 |
| 3750 | 55211 |
| 1875 | 24908 |
| 937.5 | 14908 |

**Figure 3:** Calibration curve for the glucose standard

|  |  |
| --- | --- |
| 28000 | 627164 |
| 14000 | 315765 |
| 7000 | 158955 |
| 3500 | 73225 |
| 1750 | 38407 |
| 875 | 17180 |

**Figure 4:** Calibration curve for the galactose standard

|  |  |
| --- | --- |
| 19650 | 675737 |
| 13100 | 461712 |
| 6550 | 238771 |
| 3275 | 108178 |
| 1637.5 | 63342 |
| 327.5 | 10213 |

**Figure 5:** Calibration curve for the sucrose standard

**Figure 6:** Calibration curve for the lactic acid standard

**Figure 7:** Calibration curve for the acetic acid standard

**Figure 8:** Calibration curve for the propionic acid standard

|  |  |
| --- | --- |
| 4667 | 24847977 |
| 2333.5 | 12675658 |
| 1166.75 | 6063805 |
| 583.375 | 2608173 |
| 291.688 | 1195570 |
| 145.844 | 593822 |

**Figure 9:** Calibration curve for the butyric acid standard
